# Silenced and privileged voices in media discourses: Climate change and social capital

**DOI:** 10.1371/journal.pone.0350826

**Published:** 2026-07-01

**Authors:** Maha Bashri, Sameera T. Ahmed

**Affiliations:** 1 The Africa Institute, Global Studies University, Sharjah, United Arab Emirates; 2 United Arab Emirates University, Sharjah, United Arab Emirates; PLOS, UNITED KINGDOM OF GREAT BRITAIN AND NORTHERN IRELAND

## Abstract

Media representations and narratives around climate change are often dominated by certain voices whilst others are excluded or marginalized. This study investigates media portrayal of climate change around Glasgow’s COP26, focusing on the prominence or exclusion of certain voices. Analyzing US and UK newspaper coverage, it identifies variances in representation, with Indigenous and minority voices marginalized in favor of political, scientific, and activist perspectives from the Global North. Through content analysis, the research explores how power, access, and frames shape media narratives on climate change, underscoring the need for more inclusive discussions.

## 1. Introduction

Since a relatively little-known phenomenon in the 1960s, the issue of climate change has become a part of our everyday language and continued reports of the extreme consequences of human activity on the Earth’s atmosphere and environment have become commonplace. The exclusive realm of science and research that initiated our understanding of climate change has permeated almost every facet of our lives. Much of the information that audiences obtain about related issues comes from the media [1]. Understanding who provides this information to the media is of great importance, as sources, frames, common narratives, and agendas all determine which information is conveyed (and which is omitted), how it is presented, and what impact it has on our understanding.

Most mainstream media representations of climate change stem from scientific research and government initiatives, and the terminology used to discuss issues guides how we think about and respond to them. Expressions such as ‘global warming’, ‘environmental impact’, ‘climate crises’, and ‘rising temperatures’ often obscure the fact that some people and places are affected far more than others by these phenomena. Media discourses on climate change are often dominated by perspectives, sources, and ideologies that set the agenda for how the public is informed and engages with the issue [2–4,5]. To understand the impact of sources on media coverage, this study examines selected US and UK newspapers and analyzes stories related to the 2021 United Nations Climate Change Conference of the Parties (COP26), which was held in Glasgow, Scotland. In doing so, it undertakes a quantitative and qualitative analysis of the stories in these newspapers and examines who provides information (sources and voices) and how issues are represented (frames).

By focusing on the sources cited in news articles and the themes covered in climate change stories, the study aims to uncover potential differences in representation and framing across selected media outlets. The objectives of the study are twofold: first, to analyze the types and frequencies of sources referenced in the coverage of COP26 by selected elite newspapers (*The Guardian* *(G), The New York Times* (*NYT*), and *The Washington Post* (*WP*)) and a local newspaper (*The Herald* *(H)*); and second, to identify and compare the dominant themes and frames employed by these outlets in their reporting of the event. In doing so, this study contributes to a more nuanced understanding of how media narratives on climate change are shaped by the inclusion or exclusion of certain voices and perspectives, as well as the potential implications of these narratives for public discourse and policymaking.

The paper argues that the voices of minorities and Indigenous communities, which are often the most impacted by climate crises, are the least present in the media. The terms ‘marginalized’ and ‘Indigenous’ include individuals and groups in both the Global South as well as the Global North as discrimination occurs within both spheres, though it is often more pronounced in the Global South where the impact of climate change is greater. Furthermore, it is the intersection of different types of marginalization (gender, political status, socio-economic position, etc.) which creates layers of silence in the debate about climate crises. These voices are ‘marginalised’ in that they are pushed to the periphery of climate discourse – their knowledge, experiences, and proposed solutions are less likely to be cited as sources, less likely to frame media narratives, and consequently less likely to influence public understanding and policymaking, despite their lived expertise with climate impacts. The marginalisation is not merely about numerical underrepresentation but reflects structural barriers and intersectional discrimination.

## 2. Climate change in the media: Historical overview

Media coverage on climate change and related issues is an integral aspect of news diets. Local, national, and international news organizations are increasingly dedicating more space and time to representing the causes and impacts of climate change and possible solutions. Several newspaper websites have sections dedicated to the climate crisis, and many people recognize this issue as one of the key problems in contemporary society, with their understanding facilitated mainly by the media [6–8,9]. An overview of the historical development of climate change discourse in the US media shows that references were made to the impact of human activities on the environment as long ago as the 1930s, when *The New York Times* posted that the Earth’s climate must inevitably change [10]. In the 1950s, this coverage increased somewhat, but a more noticeable increase occurred in the 1980s. Until that time, many debates took place in specialist scientific journals.

During the 1970s and 1980s, the roles of carbon dioxide, CFCs, fossil fuels, global warming, and the greenhouse effect were recognized, and these gradually became common concepts. In the mid-1980s the three spheres of media, science, and policy collided, and media coverage of climate change science and policy increased dramatically [10]. It was often key events, both natural and political, that projected the issue of climate change into the media, resulting in peaks in coverage. For example, James Hansen’s testimony to the US Congress in 1988 stated that he was 99% certain that warmer temperatures were caused by the burning of fossil fuels [11]. “Nearly everything we understand about global warming was understood by 1979. By that year, data collected since 1957 confirmed what had been known since before the turn of the 20th century: human beings have altered Earth’s atmosphere through the indiscriminate burning of fossil fuels” [12, p. 3]. While numerous scientific research studies such as ‘Changing Climate,’ the establishment of the Environmental Protection Agency (EPA), and policy initiatives such as the Clean Air Act in the USA demonstrated that climate science was beginning to impact climate policy, much work needed to be done before the public was to understand this phenomenon more accurately.

Gradually, as the relationship between science and politicians became increasingly entrenched and intermittent coverage by larger newspapers such as *The Washington Post* and *The New York Times* was joined by TV news coverage and smaller publications, public awareness began to grow. The media is now established as a key player in the relationship that began with scientists at the forefront, followed by lobbyists and politicians concerned with dealing with this global issue. In the US and many other countries, especially in the Global North, government sources, including government-funded scientists and research programs, were consulted as authorities for climate change media discourses [13]. Studies in the UK have found similar results regarding the framing of news content and its role in shaping the ways in which climate change science is presented and discussed [10,14,15]. The reliance of news media on the government and other authority figures, including scientific experts, along with journalistic practices and ideologies, has greatly influenced the framing of climate change discourses.

This, in turn, affected the public’s understanding of issues that started with a phase of awareness and developed into a move toward action in mitigating and adapting to the impact. Public attention has often turned to the climate change debate because of natural and political events. Droughts, flooding, high temperatures, hurricanes, forest fires, and similar weather events have propelled this issue into people’s minds, especially when the impact is tangible. In addition, political gatherings, such as COPs, policy initiatives, and research studies, have maintained focus on the issue in general, resulting in spikes in media attention [16]. Hase et al. [17] examined how focusing and attention-grabbing events, both unplanned and negative (extreme weather), as well as staged events (COPs), provide opportunities for some level of convergence in cross-national coverage.

### 2.1. Global South and Global North – Media narratives

Research has shown that reporting of climate crises in the Global South has both similarities and differences with reporting in the Global North. Hase et al. [17] examined media representations in ten countries from the Global South and North between 2006 and 2018; all newspapers looked at political, scientific, and societal events, which resulted in small peaks in news media attention. “However, results also indicate persistent differences: countries in the Global North covered climate change more frequently and focused more on climate science. In contrast, countries from the Global South more strongly underlined challenges and implications for society at large, especially how climate change impacts humans and their daily lives” [17, p. 10]. These differences in the focus of media narratives between the South and North are only one aspect of the unequal representation of issues related to climate change. When examining how the Global North media has represented the ongoing climate crisis, the legacy of colonialism cannot be ignored [18]. The current global situation and continuing inequality stems from centuries of colonial rule in the Global South and if current problems are analyzed without acknowledging this, it is ‘historical erasure.’ The legacy of extraction, discrimination, violence, dispossession, and imperialism has created two new forms of colonialism and racism: climate colonialism and environmental racism. Many recent examples of this can be seen in climate disasters, such as the flooding that affected Pakistan in 2022 ([19], increased farmer suicides in India, and desertification in the African Sahel [18].

Reporting in the US and UK is mostly void of any such history or context, leaving the reader with the perception that what is happening today is the result of the past few decades of changes in the climate and environment. However, these disasters are rooted in the destruction of local ecosystems, natural habitats, agricultural practices, and Indigenous community structures that occurred centuries ago during European colonialism [20,21]. Limited and negative representations of racial and minority groups and individuals are a well-established body of research in media studies [22,23,24]. Reflecting this pattern of marginalization, climate change discourses have also created an imbalance in how the public understands the topic. Just as average US citizens became concerned with CFCs and the ozone layer because it was linked to their skin cancer and had a direct impact on them, so too has much of the climate change media coverage only been concerned with how developed countries will be affected. Furthermore, in most of the analyses presented in the media, it is not made clear that the victims of climate crises are not responsible for the catastrophes they suffer [25,26].

Even when the unequal impact and injustice are identified, the movements seeking to redress these imbalances are often superficial and do not examine the root causes or historical realities of the current situation [18]. Weather and climate are blamed so that compensation, reparations, and responsibility are not on the agenda, and this discourse dominates climate action politics, permeating down to media narratives that may talk about *who* is vulnerable but not *why* they are vulnerable [15]. Sultana [27] also emphasized that controlling climate narratives by fossil fuel industries, powerful governments, and elites during COPs, as well as at other times, results in the marginalization of other voices. “Who is the expert producing climate knowledge and what expertise is generally of value to the media, policymakers, and the public? Who is setting agendas, who is invited to speak, and who is heard - often white, male experts from the Global North” [27, p. 8]. Thus, racial and ethnic minorities, Indigenous communities, and marginalized groups, including women, are underrepresented in policy formation, academia, and the media.

### 2.2. Voices – Silenced and privileged

Considerable evidence confirms that the groups or communities that are directly affected by the worst instances of climate change are the least responsible for it [28]. However, compared to 10 or 20 years ago, and especially with the advent of the World Wide Web, minority voices have been able to create platforms for representing themselves and issues that concern them. The impact of this on mainstream discourse is not clear; even when the plight of Indigenous people and minority groups is recognized by the mainstream media, they are often spoken of or about rather than spoken with or to, which prevents them from articulating their own experiences and perspectives in their own voices [29]. Over time, the predominance of governmental and scientific voices has declined, and local activists, Indigenous Groups, campaigners, and environmental NGOs have joined the conversations about climate crises. There has been an evident increase in sources and voices speaking about climate change, climate justice, and climate action across different types of media. However, while different voices work toward the same goal, they do not exert the same power as western governments [30]. In addition, the discursive structures within which knowledge, actions, solutions, and financing of climate change are framed or subsumed reflect Western hegemony [27] whereby international governmental bodies headquartered in industrialized and economically developed nations dictate how to deal with the climate crisis.

The unequal footing on which different stakeholders concerned with the climate crisis operate is manifest in news media practices. Certain voices and sources are privileged, especially if they fit into existing frames of reference and help reinforce the predominant discourse [31,32,33]. Other voices are marginalized or totally excluded, and if they are given space, it is often to underpin pre-existing notions and stereotypes or to help bolster current ideologies. “Thus, media coverage of climate change adaptation and mitigation is not a simple collection of news articles and clips produced by journalists and producers. Rather, representations signify key frames derived from the complex and nonlinear relationships between scientists, policy actors, and the public, often mediated by news stories” [10, p. 9]. Narratives that dominate climate science and policy determine media discourse, which subsequently affects the public’s understanding of these issues.

As our understanding of and engagement with climate change issues increase, it becomes vital to appreciate how this has developed and what impact it can have on our perceptions and responses to events unfolding in our localities and around the world. Context and history are as important as equal and fair representation in contemporary media debates [34]. While there is recognition in certain news organizations that diversity and critical voices are essential in news about climate change, it remains to be seen how this will manifest in practice. This study contributes to examining the presence of these ‘silenced voices’ in media debates about climate crises.

## 3. Social capital and media access in climate discourse

Social capital, as conceptualized by Bourdieu [35], operates as symbolic currency that individuals and institutions accumulate through networks, relationships, and social positioning. In media contexts, social capital manifests as differential access to journalists and platforms for public discourse [36,37]. This becomes particularly crucial in climate reporting, where competition for media attention among diverse stakeholders determines whose perspectives shape public understanding.

Social capital operates in media contexts through three key mechanisms. First, institutional proximity grants certain actors privileged access through formal channels. Government agencies, scientific institutions, and large NGOs maintain press offices and established communication protocols that facilitate regular interaction with news organizations, creating what Gans [38] describes as symbiotic relationships between journalists and official sources [39].

Second, professional credibility functions as cultural capital that translates into media access [40]. Sources with recognized expertise, academic credentials, or official positions become attractive to journalists seeking to demonstrate reliability and authority. This creates systematic advantages for elite institutional actors over grassroots voices.

Third, network embeddedness creates ongoing relationships that extend beyond individual stories. Elite sources often move between government, academia, and private sector positions while maintaining journalist contacts. These networks create what Coleman [36] identifies as social closure which is information circulation among established members while excluding outsiders.

Marginalized communities face systematic barriers reflecting broader social inequalities. Indigenous communities, racial minorities, and Global South organizations typically lack institutional infrastructure for consistent media engagement [29,41]. The temporal dynamics of news production further disadvantage low social capital actors, as journalists under deadline pressure turn to sources providing immediate, authoritative responses [29]. Moreover, the linguistic and cultural capital required for effective media engagement reflects dominant cultural norms that may alienate marginalized communities whose knowledge systems operate within different frameworks. This creates what Couldry [42] terms media capitals; differential abilities to engage effectively with media institutions.

Journalistic practices themselves reproduce social capital inequalities. The beat system creates ongoing relationships with official sources while potentially limiting exposure to alternative perspectives [43]. The verification imperative advantages sources whose credentials can be quickly confirmed through official channels, systematically disadvantaging those whose knowledge comes from lived experience rather than institutional affiliation [44].

In climate discourse specifically, high social capital actors (particularly in the Global North) often frame climate change as a technical problem requiring technological solutions, aligning with elite institutional positions while potentially obscuring justice dimensions emphasized by marginalized communities [3,45]. This concentration of narrative control means that community-based solutions, Indigenous knowledge systems, and grassroots resilience strategies may remain invisible in mainstream discourse despite their effectiveness [26].

The climate change narrative, as represented by elite and local media during COP26, provides a compelling lens through which to explore the power dynamics of social capital. Additionally, it illuminates the influence of historical precedence on source selection and the broader implications this has on framing. In pursuing a deeper understanding of the media dynamics surrounding climate change coverage during COP26, this study aimed to scrutinize the following research questions:

*RQ1: What dominant frames are used in the news coverage of climate change during COP26 by the four newspapers (The Guardian, The New York Times, The Washington Post, and The Herald)?*

*RQ2: How do the types and frequencies of news sources cited in the coverage of climate change during COP26 compare among the four newspapers?*

*RQ3: How does the representation of climate change differ among the four newspapers?*

## 4. Methodology

This study investigated media portrayals of and reactions to COP26 in Glasgow during 2021. Recognizing the event's significant role in influencing public discourse, the analysis centered on the conference's first week from October 31 to November 6, 2021. This study integrated quantitative and qualitative methodologies whereby potential statistical correlations between the themes, referenced sources and voices, and the solutions offered in the news outlets’ coverage were explored. In parallel, qualitative content analysis was conducted to uncover the depth, patterns, and emerging themes. This dual approach combined the depth of qualitative insights with the empirical rigor of quantitative correlations.

### 4.1. Sample selection

To gain a multifaceted perspective, articles were selected from four leading news outlets: *NYT*, *WP*, *G*, and *H*. These outlets, with their distinct editorial voices, offer both global and regional insights on issues relating to climate. Furthermore, the *NYT* has been a pioneer in articulating concerns about climate change and in the UK, the *G* has been the leading newspaper to engage in debates about climate crises. The *H* is the local newspaper of Glasgow and is closest to the event, offering a local perspective and having access to all COP26 participants. The *WP* is deemed to be a bellwether for policy direction in the US.

Articles were sourced using the Gale OneFile Complete database, using specific keywords: “COP26”, “Glasgow COP”, “Glasgow 2021”, and “2021 United Nations Climate Change Conference.” Emphasis was placed on hard news stories, excluding opinion pieces and editorials. The initial search yielded between 70 and 90 articles per outlet. To ensure methodological coherence and enable valid cross-newspaper comparisons, we standardized the dataset by selecting 50 articles from each publication through systematic random sampling. From each outlet's available articles, we numbered all articles chronologically and selected every nth article (where n = total articles/50) to achieve our target sample of 50 articles per newspaper, yielding a total corpus of 200 articles. This approach ensured both temporal representation across the week and proportional coverage from each outlet.

Any debate on climate change will focus on three main aspects: causes, consequences, and solutions, from scientific and public perspectives. Therefore, these primary categories were included in the analysis and supplemented by supporting themes identified from the literature, specifically Rich [12], and historical discourses on climate change.

Articles were coded for the institutional affiliation or role of sources cited, including: politicians/government officials, scientists/scientific studies, grassroots activists, NGO representatives, business/corporate sources, and Indigenous community leaders.

To capture representation of voices typically excluded from climate discourse, we coded whether articles included perspectives from: Indigenous groups, women and girls, youth and young people, Global South communities, and other marginalized populations. Sources could be coded in both schemes simultaneously (for example, an Indigenous activist would be coded both as “grassroots activist” under primary sources and as “Indigenous groups” under marginalized voices). Source categorization followed a role-based coding protocol determined by how media outlets positioned each source within the article. When individuals held multiple identities (for example, Vanessa Nakate as young, female, and Ugandan), coding reflected the specific role attributed to them by the journalist in that context. If an article quoted Nakate discussing youth-led climate movements, she was coded as youth; if quoted as a protest organizer, she was coded as grassroots activist. This approach aligns with the study's focus on media representation: rather than coding sources according to all their demographic characteristics, we coded according to the role the media assigned them, capturing how journalists construct and deploy different voices in climate discourse. This role-based approach ensures the analysis reflects media framing practices rather than imposing external categorizations onto sources.

For this study, marginalized voices refer to groups that face systematic barriers to media access and representation in climate discourse, typically lacking the institutional proximity, professional credibility, and network embeddedness that facilitate routine media access [27,29]. We coded for five categories: Indigenous groups, women and girls, youth and young people, Global South communities, and other marginalized populations facing intersecting forms of exclusion. Details of the specific codes within these categories are provided in S1 Appendix.

A comprehensive codebook was devised to guide the systematic analysis of all articles. Initially, four coders independently assessed a subset constituting 10 percent of the total number of articles (20 articles) to establish intercoder reliability. Two research assistants were recruited to code newspaper articles using selected themes. Discrepancies were addressed through discussion, and the codebook was refined accordingly. The inter-coder reliability across all categories was 0.87, measured using Krippendorff's alpha. This rate of intercoder reliability ensured significant accuracy and consistency when coding the same content.

### 4.2. Analytical approach

The qualitative component of this study employed inductive thematic analysis following Braun and Clarke’s [46] widely cited framework. Unlike deductive approaches that apply pre-existing theoretical categories to data, inductive thematic analysis allows themes to emerge directly from the data itself, with codes and patterns strongly linked to the data themselves rather than predetermined by the researchers’ analytic preconceptions [46]. This approach is particularly suited to exploratory research examining how media construct climate change narratives, as it enables identification of patterns that might otherwise be obscured by imposing external frameworks.

Theme development followed an iterative, six-phase process [46,47]. First, researchers engaged in familiarization through repeated reading of all 200 articles to develop intimacy with the data. Second, initial coding involved systematic line-by-line examination of articles, generating codes that captured semantic content related to sources, frames, and representations. Third, during the searching for themes phase, related codes were collated into potential thematic categories. Fourth, themes underwent review to ensure internal coherence and external distinctiveness—themes that lacked sufficient data support were either collapsed into related categories or, where they appeared only once or twice across the dataset without connecting to broader patterns, were categorized as Other. Fifth, themes were defined and named to capture their essential character. Sixth, findings were synthesized into the final analysis.

The Other category warrants methodological explanation. In inductive thematic analysis, not all coded content coalesces into robust themes; some frames appeared sporadically (one or two instances) and did not demonstrate sufficient prevalence or patterning to constitute standalone themes [48,49]. Rather than forcing these isolated frames into ill-fitting categories or discarding them entirely, we retained them under Other to preserve analytic transparency. This approach aligns with qualitative research principles that prioritize the characteristics and meaning of concepts reflected in the data over mere frequency of occurrence [50]. Examples of content categorized as Other included local public, police, emergency services (source); travel to COP, wheelchair access, speeches (theme); destruction of habitats, loss of culture, animals (consequences); greenwashing, lack of transparency, disagreement about facts/numbers (conflict); NGOs, community organisations (voices); lack of investment, lack of awareness, resource allocation (causes); and technology, parity and justice, industry/corporate behaviour (solutions).

## 5. Findings

### 5.1. RQ1: What dominant frames are used in the news coverage of climate change during COP26 by the four newspapers?

In examining the frames employed by the four news outlets, we found a nuanced landscape of journalistic priorities. Each outlet demonstrated its own emphases, though common threads weave through their reporting styles. A chi-square test revealed a statistically significant association between publication type and thematic framing (χ²(27) = 90.258, p < 0.001) (Table 1).

**Table 1:** Distribution of themes/frames across publications.

	The Guardian	The Herald	Washington Post	New York Times	
*Category*	*N (%)*	*N (%)*	*N (%)*	*N (%)*	*Total N*
Emergency/Crisis/Warning	14 (20.9)	8 (12.7)	9 (11.5)	31 (27.0)	62
Protest	1 (1.5)	21 (33.3)	5 (6.4)	16 (13.9)	43
Govt Resistance/Inaction	8 (11.9)	9 (14.3)	11 (14.1)	10 (8.7)	38
Criticism/Critique	16 (23.9)	3 (4.8)	12 (15.4)	22 (19.1)	53
Frustration/Anger/Pessimism	3 (4.5)	5 (7.9)	17 (21.8)	6 (5.2)	31
Misinformation	1 (1.5)	0 (0.0)	2 (2.6)	0 (0.0)	3
Unequal Impact	6 (9.0)	1 (1.6)	0 (0.0)	8 (7.0)	15
Discrimination/Marginalization	1 (1.5)	0 (0.0)	1 (1.3)	2 (1.7)	4
Climate Colonialism	3 (4.5)	0 (0.0)	0 (0.0)	0 (0.0)	3
Other	14 (20.9)	16 (25.4)	21 (26.9)	20 (17.4)	71
***Total mentions***	***67***	***63***	***78***	***115***	***323***

Note. χ²(27) = 90.258, p < 0.001. Multiple themes could be coded per article (total coded mentions = 323). Column percentages shown in parentheses.

*G* offered a balanced approach to news coverage, with notable attention to “emergency, crisis, or warning” frames alongside coverage of “government resistance, inaction, or funding” and “protest” themes. Significantly, a substantial portion of its articles addressed themes categorized as “other,” indicating a wide scope of reporting beyond predetermined categories. This distribution suggests that *G* maintains versatility in its news coverage while remaining attuned to urgent matters requiring immediate public attention.

In contrast, *H* demonstrated a pronounced focus on civil society matters and grassroots movements. The outlet allocated the highest proportion of its coverage to “protest” themes among all four newspapers, while also featuring a large portion under the “other” category. This pattern suggests *H* concentrated on civil society issues and covered topics that did not fit comfortably within other defined themes. The newspaper demonstrated unique attention to intersecting themes of emergency, protest, and governmental responses, suggesting specialized reporting where these issues converge.

The *WP's* coverage pattern differed notably, with distinctive attention to “frustration, anger, or pessimism” frames—receiving more emphasis here than in other outlets. The outlet also covered “government resistance, inaction, or funding” and “criticism or critique” frames, while allocating a substantial portion to “other” themes. This pattern suggests the *WP* prioritized capturing public sentiment and emotional dimensions of the climate debate alongside policy discussions.

*NYT* placed notably greater emphasis on analytical frames, with relatively high coverage devoted to “criticism or critique” and “unequal impact” themes compared to other outlets. This suggests that the outlet prioritized analytical and social justice-oriented angles in its reporting, in addition to covering immediate news events. The newspaper also offered diversified thematic coverage with a considerable portion of articles categorized as “other.”

While all four outlets dedicated substantial portions of their coverage to themes falling outside predetermined categories, each demonstrated distinctive editorial emphases. *G* leaned toward balanced and urgent reporting, *H* focused intensively on civil society and protest, the *WP* attended closely to societal sentiments and emotional responses, and *NYT* adopted a more analytical and socially conscious approach. These findings highlight the diverse landscape of news coverage, where each outlet offers a specialized lens through which audiences understand the complexities of climate change, shaped by their readership's needs and ideological positioning.

### 5.2. Solutions framing

**Fig 1:**
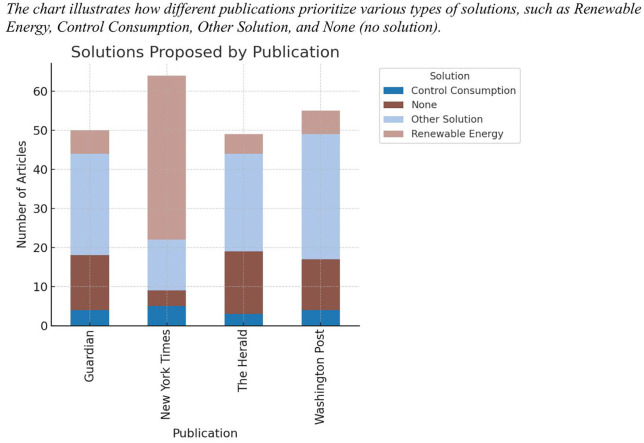
Distribution of climate change solutions across publications. Stacked bar chart showing the frequency of solution types (renewable energy, controlling consumption, other, none) by newspaper. Column heights represent total coded mentions per outlet.

We investigated the relationship between publications and the climate change solutions they advocated for or discussed. The chi-square test revealed a highly significant relationship between publication type and solutions presented (χ²(9) = 75.630, p < 0.001) (Table 2, Fig 1).

**Table 2:** Distribution of climate change solutions across publications.

	The Guardian	The Herald	Washington Post	New York Times	
*Category*	*N (%)*	*N (%)*	*N (%)*	*N (%)*	*Total N*
Renewable Energy	6 (11.8)	6 (11.1)	6 (10.9)	42 (66.7)	60
Controlling Consumption	4 (7.8)	3 (5.6)	4 (7.3)	5 (7.9)	16
Other	27 (52.9)	27 (50.0)	32 (58.2)	12 (19.0)	98
None	14 (27.5)	18 (33.3)	13 (23.6)	4 (6.3)	49
***Total mentions***	***51***	***54***	***55***	***63***	***223***

Note. χ²(9) = 75.630, p < 0.001. Multiple solutions could be coded per article (total coded mentions = 223). Column percentages shown in parentheses.

*NYT* demonstrated a pronounced focus on renewable energy solutions, featuring this solution type far more extensively than other newspapers in the sample. By contrast, *G* presented a more diverse portfolio of solutions, ranging from renewable energy to consumption control strategies, leading among outlets in attention to consumption-focused approaches. *H* and *WP* both adopted more balanced approaches, featuring relatively equal distributions across renewable energy, consumption control, and other solution types. Notably, the *WP* and *H* together provided substantial attention to solutions categorized as ‘Other,’ suggesting coverage of alternative or innovative approaches not captured by the predetermined solution categories.

An important pattern emerged in *H's* coverage: while it extensively addressed various themes including protests and local impacts, it offered limited explicit discussion of concrete climate change solutions. The outlet acknowledged the climate crisis within the context of COP26 but did not examine practical mitigation or adaptation strategies in substantive detail, instead focusing primarily on documenting the event itself and community responses.

### 5.3. RQ2: How do the types and frequencies of news sources cited in the coverage of climate change during COP26 compare among the four newspapers?

**Fig 2:**
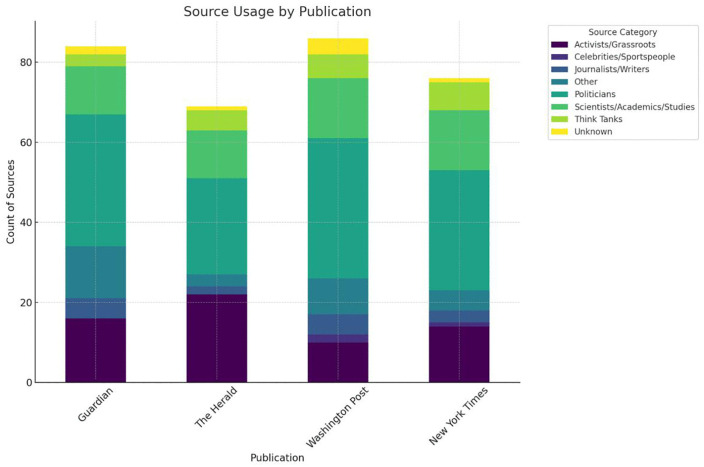
Distribution of sources across publications. Stacked bar chart showing the frequency of source categories (politicians, scientists/academics, activists/grassroots, journalists/writers, celebrities/sportspeople, think tanks, other, unknown) by newspaper.

The analysis examined source utilization patterns across the four publications. A chi-square test did not reveal a statistically significant association between publication type and source type (χ²(18) = 21.641, p = 0.248) (Table 3, Fig 2). While descriptive differences in source utilization were observed across publications, these did not reach statistical significance, suggesting a structural homogeneity in sourcing practices that transcends individual outlet characteristics. Nevertheless, the descriptive patterns are instructive.

**Table 3:** Distribution of sources across publications.

	The Guardian	The Herald	Washington Post	New York Times	
*Category*	*N (%)*	*N (%)*	*N (%)*	*N (%)*	*Total N*
Politicians	33 (40.2)	25 (33.3)	35 (42.7)	30 (40.0)	123
Scientists/Academics	12 (14.6)	16 (21.3)	15 (18.3)	15 (20.0)	58
Activists/Grassroots	16 (19.5)	23 (30.7)	10 (12.2)	14 (18.7)	63
Journalists/Writers	5 (6.1)	3 (4.0)	5 (6.1)	3 (4.0)	16
Celebrities/Sportspeople	0 (0.0)	0 (0.0)	2 (2.4)	1 (1.3)	3
Think Tanks	3 (3.7)	5 (6.7)	6 (7.3)	7 (9.3)	21
Other	13 (15.9)	3 (4.0)	9 (11.0)	5 (6.7)	30
***Total mentions***	***82***	***75***	***82***	***75***	***314***

Note. χ²(18) = 21.641, p = 0.248. Result not statistically significant. Multiple sources could be coded per article (total coded mentions = 314). Column percentages shown in parentheses.

### 5.4. Primary source patterns

Examination of source types across the four publications revealed both expected patterns and surprising contradictions. Although the overall distribution of source types did not differ significantly across outlets, notable descriptive patterns emerged. Political sources- government officials, politicians, and policy leaders -featured prominently across all newspapers, though at varying levels. *G* demonstrated the highest reliance on political sources among the four outlets, followed closely by *NYT*. Somewhat contrary to expectations about policy-focused outlets, the *WP* cited politicians somewhat less frequently than these progressive newspapers.

*NYT*, despite its left-leaning reputation, relied substantially on politicians as primary sources. However, the outlet also drew extensively from diverse sources including academics, activists, and marginalized communities, which it deployed strategically to highlight the severity and multifaceted nature of the climate crisis.

*H*, as a local publication, distinguished itself through different source priorities. The newspaper prominently featured grassroots and activist sources, leading all outlets in this category. This emphasis likely reflects the newspaper's proximity to COP26 and direct access to activists and community organizers present at the Glasgow summit. Additionally, *H* cited scientists and scientific studies extensively, reflecting access to researchers and experts participating in the conference.

The *WP* also demonstrated notable attention to scientific sources alongside its political coverage, indicating commitment to incorporating scientific expertise into reporting.

### 5.5. Representation of marginalized voices

**Fig 3:**
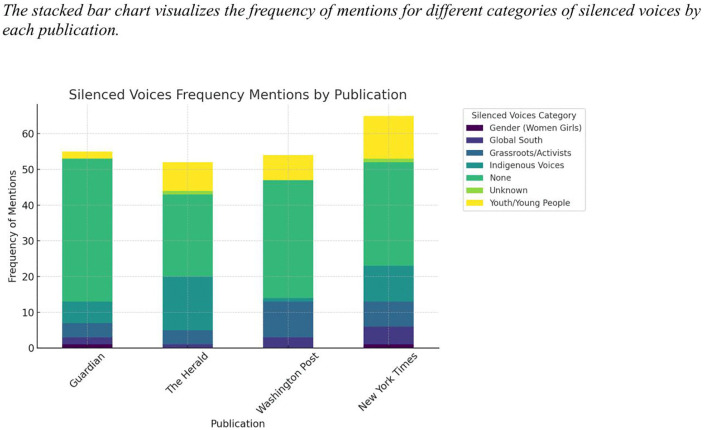
Distribution of silenced/marginalized voices across publications. Stacked bar chart showing the frequency of marginalized voice categories (gender, Global South, Indigenous groups, youth/young people, grassroots/activist, none) by newspaper.

A separate analysis examined how publications represented what we term “silenced voices”—perspectives from communities typically marginalized in climate discourse. This analysis revealed a statistically significant relationship between publication type and marginalized voice representation (χ²(15) = 32.314, p = 0.006) (Table 4, Fig 3).

**Table 4:** Distribution of silenced/marginalized voices across publications.

	The Guardian	The Herald	Washington Post	New York Times	
*Category*	*N (%)*	*N (%)*	*N (%)*	*N (%)*	*Total N*
Gender (Women/Girls)	0 (0.0)	0 (0.0)	0 (0.0)	1 (1.6)	1
Global South	2 (3.8)	2 (3.6)	3 (5.6)	5 (7.8)	12
Indigenous Groups	5 (9.4)	15 (26.8)	1 (1.9)	10 (15.6)	31
Youth/Young People	2 (3.8)	8 (14.3)	7 (13.0)	12 (18.8)	29
Grassroots/Activist	4 (7.5)	4 (7.1)	10 (18.5)	7 (10.9)	25
None	40 (75.5)	27 (48.2)	33 (61.1)	29 (45.3)	129
***Total mentions***	***53***	***56***	***54***	***64***	***227***

Note. χ²(15) = 32.314, p = 0.006. Multiple voice categories could be coded per article (total coded mentions = 227). Column percentages shown in parentheses.

Within the subset of articles that included marginalized voices, distinct patterns emerged across the four newspapers:

The *H* led substantially in providing platform space to Indigenous groups. Among articles featuring Indigenous perspectives across all four newspapers, *H* published the clear majority of such coverage. This pronounced focus likely reflects the newspaper's proximity to COP26 and the significant presence of Indigenous delegations at the Glasgow summit.

Surprisingly, the *WP* provided the most coverage of youth and young people's perspectives among the four outlets, accounting for most of the youth-focused coverage across the sample. This finding challenges assumptions that youth climate voices would be most prominent in more progressive outlets.

The *G* predominantly featured grassroots and activist voices among the publications, consistent with its progressive editorial stance and historical attention to social movements. However, *H* also strongly emphasized grassroots sources, reflecting its local embeddedness and access to activists present in Glasgow.

A notable gap emerged in the *G'*s coverage: despite its progressive reputation and emphasis on grassroots voices, it included minimal articles specifically highlighting women and girls as distinct voices in climate discourse. The other outlets provided modest but present representation of gendered perspectives in their coverage.

These findings reveal complex patterns that challenge straightforward assumptions about ideological orientation and source diversity. Elite newspapers, regardless of political leaning, demonstrated significant reliance on political sources, while the local outlet distinguished itself through greater attention to grassroots and Indigenous voices. Within marginalized voice representation, each outlet demonstrated distinct priorities that did not align predictably with their general reputations.

### 5.6. RQ3: How does the representation of climate change differ among the four newspapers?

Comparative analysis revealed substantial differences in how the four newspapers approached climate change representation, differences extending beyond simple source selection to encompass narrative focus, thematic emphasis, and geographical scope.

The *H's* coverage of COP26 distinguished itself through intensive focus on local and grassroots narratives. Unlike its elite counterparts, *H* explored in substantial detail the impacts of climate change on local communities and Indigenous groups, bringing stories and perspectives typically overlooked by national and international outlets to the forefront. This localized reporting provided not merely a unique angle on a global issue but highlighted the diversity of experiences and responses to climate change across different community contexts.

The newspaper's proximity to the summit enabled direct access to diverse participants, particularly Indigenous delegates and grassroots activists who traveled to Glasgow. This access translated into coverage that prioritized voices from affected communities over policy pronouncements from national leaders. Where *H* differed most markedly from elite outlets was in its relative de-emphasis on solutions: the newspaper documented the climate crisis and community responses extensively but offered limited analysis of concrete mitigation or adaptation strategies, focusing instead on experiential accounts and protest movements.

*G*, *NYT*, and *WP* demonstrated convergent approaches despite their geographical and ideological differences. All three elite outlets prioritized political, economic, and scientific angles in their climate change coverage. Their reporting frequently centered on international agreements, political debates between world leaders, economic implications of climate action, and scientific studies. These newspapers relied predominantly on official sources—politicians, scientists from established institutions, and international organizations—to provide top-down perspectives on climate-related issues.

However, important distinctions existed among the elite outlets:

*G* balanced scientific and political coverage with strong attention to activist perspectives and protest movements. Its emphasis on emergency and warning frames reflected editorial commitment to conveying urgency. However, its limited attention to gendered perspectives suggests gaps in its approach to representing marginalized voices.

*NYT* distinguished itself through greater emphasis on analytical and social justice frames. Its relatively high focus on unequal impact and critical analysis positioned it as providing more contextual examination of climate justice dimensions. Its strong focus on renewable energy solutions indicated clear editorial prioritization of technological pathways to climate mitigation.

*The WP* demonstrated unique attention to emotional and sentiment dimensions of climate discourse, with substantial coverage framed around frustration, anger, and pessimism. This suggests editorial recognition that public mood and emotional responses constitute important dimensions of climate politics. Its leadership in representing youth voices aligned with attention to emerging movements and generational perspectives.

Several patterns emerged across all publications. First, substantial portions of coverage addressed themes outside predetermined categories, suggesting that climate discourse during COP26 encompassed diverse topics not easily captured by conventional framing categories. Second, all outlets demonstrated some attention to marginalized voices, though in different proportions and with different emphases. Third, political sources featured prominently across all newspapers, reinforcing that institutional political actors maintain privileged access to media representation regardless of outlet ideology.

## 6. Discussion: Contrasting media narratives and implications for future climate discourse

Social capital theory provides an interpretive framework for understanding the source patterns identified through content analysis. While the three mechanisms; institutional proximity, professional credibility, and network embeddedness; operate within journalistic production processes that this study does not directly observe, their effects manifest in the differential representation of voices documented in our findings.

This study reveals both expected patterns and surprising contradictions in climate coverage during COP26, offering insights into how ideology, geography, and structural constraints shape media narratives.

The most striking finding challenges assumptions about progressive media and source diversity. Both progressive outlets, *G* and *NYT*, relied heavily on politicians as primary sources, with *G* citing political sources most frequently among all four outlets. The centrist *WP* cited politicians less frequently, while the local newspaper *H* led all outlets in grassroots source citations—contradicting expectations that elite outlets provide greater diversity.

Progressive ideology of media outlets does not automatically translate into source diversity or representation of marginalized voices and geographic proximity significantly influences coverage that privileges elite sources across ideological boundaries. Across the ideological spectrum, mainstream media demonstrate common reliance on the same “news definers”: political authorities, established scientific institutions, and recognized experts [51]. Indeed, the chi-square analysis found no statistically significant differences in source type distribution across the four outlets (p = 0.248), confirming that sourcing practices are structurally consistent regardless of editorial orientation. This convergence suggests that journalistic practices, professional norms, and structural constraints around access exert stronger influence on source selection than editorial ideology alone [52].

This finding carries critical implications: calls for more diverse climate coverage cannot rely solely on supporting progressive outlets, as they too reproduce elite source dominance. Structural changes in journalistic practice, actively seeking marginalized voices, dedicating resources to grassroots movements, and countering default reliance on official sources, are necessary regardless of outlet ideology.

*H*'s distinctive coverage illuminates how geographic proximity shapes representation. As Glasgow's local outlet, *H'*s intensive focus on Indigenous voices and grassroots activists reflects direct access to COP26 participants that elite national outlets, despite greater resources, largely overlooked. This suggests local media play crucial roles in amplifying marginalized voices during major international events. Their embeddedness in host communities positions them to provide representation that supplements or challenges elite media's top-down narratives. However, *H*'s limited attention to concrete solutions reveals potential limitations: local outlets may excel at documenting presence and protest but lack resources to analyze complex policy mechanisms or technical solutions.

Despite some representation of marginalized voices, persistent exclusion patterns emerge. *G'*s minimal gender-focused coverage stands out given its progressive reputation and stated commitment to diverse perspectives, suggesting even social justice-oriented outlets maintain blind spots around intersectional dimensions of climate vulnerability.

More broadly, Indigenous, youth, and Global South perspectives remained proportionally limited across all outlets, indicating marginalized voices remain marginal in elite climate discourse. When these voices appear, they are often deployed strategically, to illustrate impact or represent protest, rather than as authoritative sources on solutions, policy, or science. This reflects Sultana's [27, p. 8] observation: expertise valued by media and policymakers typically comes from “white, male experts from the Global North.” Our findings confirm this dynamic operates even in progressive outlets during a summit where Indigenous and Global South delegates were prominently present.

*NYT*'s overwhelming focus on renewable energy suggests a primarily technological framing that may crowd out equally important approaches: consumption reduction, structural economic change, climate justice and reparations, or adaptation strategies for vulnerable communities. *G*'s more diverse solution portfolio, encompassing both renewable energy and consumption control, indicates recognition that technological fixes alone prove insufficient. However, even this approach may inadequately represent solutions prioritized by Indigenous communities and Global South nations—approaches centered on traditional ecological knowledge, community-led adaptation, and climate justice demands rather than purely technical or market-based mechanisms.

*H*'s relative absence of solution-focused coverage is noteworthy: while extensively documenting protest and community presence, the newspaper offered limited analysis of concrete pathways forward, suggesting limitations in local outlets’ capacity to bridge grassroots representation and policy analysis.

These findings align with the theoretical framework of social capital and media access. Sources with greater social capital—political institutions, established scientific organizations, wealthy nations—enjoy privileged media access. Their perspectives disproportionately shape how climate issues are framed, which problems receive attention, and which solutions appear viable. The information they provide, influenced by their own interests (policy priorities, scientific paradigms, economic considerations), dominates coverage and public discourse.

Conversely, those with limited social capital—Indigenous communities, Global South nations, grassroots movements—have reduced media access, are sought less frequently by journalists, and see their experiences receive less attention and carry less authority. How the public perceives climate issues depends fundamentally on this unequal relationship. When renewable energy solutions dominate coverage while climate reparations receive minimal attention, when political debates between wealthy nations take center stage while Pacific Island communities remain marginal, public understanding inevitably reflects and reinforces existing power hierarchies. The findings challenge assumptions about progressive media: progressive outlets don't automatically provide more diverse representation; geographic proximity significantly influences coverage; and elite source dominance persists across ideological boundaries.

This study has three main limitations. First, examining only Global North newspapers misses Global South perspectives on COP26 coverage. Second, analyzing only the summit's first week may overlook narrative evolution during the second week when negotiations intensified and agreements were reached. Third, the 50-article sample per outlet, while enabling valid comparisons, represents only a subset of available coverage; larger samples might reveal additional patterns. Further research could also examine social media discourses along with those in traditional, mainstream outlets providing an important comparative analysis.

Social media platforms have introduced opportunities for marginalized voices to gain visibility. Their relatively open access and participatory nature provide spaces for non-traditional actors to accumulate alternative forms of social capital [53]. Grassroots activists, Indigenous communities, and those most affected by climate change can share perspectives, experiences, and solutions directly [54]. Movements like Fridays for Future and the Indigenous Environmental Network have capitalized on social media to shift climate discourse, emphasizing urgency and foregrounding equity and justice demands [55].

However, this study's findings suggest social media's impact on mainstream media representation remains limited. While enabling marginalized voices to build movements and reach audiences directly, elite outlets largely maintain traditional sourcing patterns privileging official voices. The challenge involves understanding how alternative platforms can more effectively pressure mainstream media to expand source bases and adopt more inclusive framing practices.

Several practical approaches can increase diverse sources in climate debates. Elite media outlets must partner with community organizations, Indigenous networks, and grassroots movements—not merely as subjects but as collaborators in shaping coverage. Alignment between mainstream and alternative media, integrating social media, user-generated content, and citizen journalism, could enable silenced voices to articulate climate impacts and offer solutions grounded in Indigenous knowledge and grassroots activism rather than exclusively in political deliberations and scientific institutions.

This requires structural newsroom changes: dedicating beats to environmental justice rather than only environmental science, recruiting journalists from marginalized communities, and developing relationships with sources beyond usual official channels. Media organizations can mitigate social capital imbalances by actively seeking and amplifying voices from underrepresented regions and communities. This extends beyond token representation in human interest stories to positioning marginalized voices as authoritative sources on policy, solutions, and scientific knowledge—recognizing that those most affected often possess crucial expertise about adaptation, resilience, and justice that elite sources lack.

Achieving more inclusive climate discourse requires structural transformation of journalistic practices, not simply relying on presumably progressive outlets. Media must intentionally cultivate relationships with marginalized communities and recognize diverse forms of expertise beyond traditional institutions. Only through such changes can climate coverage adequately represent those most affected, center the justice dimensions essential to addressing this crisis and work towards redressing the existing imbalance of social capital. Considering the power of the media to affect not only public perception but potentially policy and decision making at national and global levels, these recommendations can be seen as part of a broader framework within which we understand and respond to climate change. The relationship between those with greater social capital (governments, politicians, policy makers, powerful corporations etc.) and media remains strongly intertwined but disrupting this entrenched flow of information and opening up the debate to voices with different perspectives and experiences will benefit us as individuals and societies and have a positive impact on how we mitigate and manage the impact of climate change.

## Supporting information

S1 Appendix Variable Values. (DOCX)
