# Supplementary figures and images for: Silenced and privileged voices in media discourses: Climate change and social capital
Source: PLoS One. 2026 Jul 1;21(7):e0350826. doi: 10.1371/journal.pone.0350826 (PMC13322496; doi:10.1371/journal.pone.0350826)

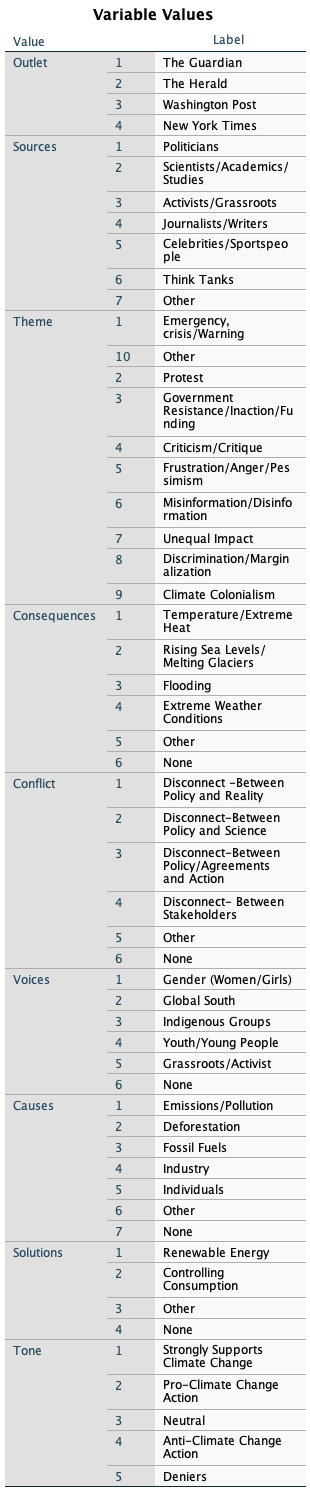

Supplement: S1 Appendix — (DOCX) [file pone.0350826.s001.docx]
