# Supplementary material for: Silenced and privileged voices in media discourses: Climate change and social capital
Source: PLoS One. 2026 Jul 1;21(7):e0350826. doi: 10.1371/journal.pone.0350826 (PMC13322496; doi:10.1371/journal.pone.0350826)
Supplement: Attachment — Submitted filename: Response to Reviewers.docx [file pone.0350826.s002.docx]

**RESPONSE TO REVIEWERS**

# REVIEWER #1

Dear Dr. Najm A. Alhatimi Aleessawi,

We are grateful for the time, care, and scholarly rigor you brought to your review of our manuscript. Your comments reflect a deep engagement with both the methodological and theoretical dimensions of the study, and we found your observations to be incisive and constructive. We have addressed each of your points below, with specific references to where revisions appear in the text.

**1. Methodological Enhancements and Transparency**

***Concern:*** *The manuscript fails to detail the specific qualitative framework employed.*

**Response:** Our study employed inductive thematic analysis following Braun and Clarke’s (2006, 2021) frameworka well-established approach for media content research. To enhance clarity, we have added a dedicated *Analytical Approach* subsection to the methodology that details the six-phase process of theme development: familiarization, initial coding, searching for themes, reviewing themes, defining themes, and final synthesis.

***Concern:*** *The “Other” category must be broken down or summarized.*

**Response:** The “Other” category represents frames that appeared only once or twice across the dataset and did not demonstrate sufficient patterning to constitute standalone themes. Retaining these under “Other” preserves analytic transparency rather than forcing isolated instances into ill-fitting categories. To address the reviewer’s concern, we have added concrete examples: content categorized as Other included local public, police, and emergency services (sources); travel logistics, wheelchair access, and speeches (themes); destruction of habitats, loss of culture, and impacts on animals (consequences); greenwashing, lack of transparency, and disagreement about facts (conflict); NGOs and community organisations (voices); lack of investment, awareness, and resource allocation (causes); and technology, parity and justice, and industry behaviour (solutions).

***Concern:*** *Provide raw coding data as supplementary files.*

**Response:** The raw coding data has been deposited in a public repository. To preserve blind review, the DOI and access details have been provided to the editor and will be included in the published manuscript upon acceptance. The dataset has also been shared with the editor. Additionally, we have now embedded Tables 1–4 containing the full chi-square cross-tabulations in the manuscript.

**2. Clarification of Source Categorization**

***Concern:*** *The manuscript requires a more precise definition of marginalized voices.*

**Response:** We have added a definition in the methodology section. For this study, *marginalized voices* refers to groups that face systematic barriers to media access and representation in climate discourse, typically lacking the institutional proximity, professional credibility, and network embeddedness that facilitate routine media access. These voices are marginalized in that they are pushed to the periphery of climate discoursetheir knowledge, experiences, and proposed solutions are less likely to be cited, less likely to frame media narratives, and consequently less likely to influence public understanding and policymaking, despite their lived expertise with climate impacts.

***Concern:*** *Clarify the coding hierarchy when an individual fits multiple categories.*

**Response:** Our coding followed a role-based protocol consistent with the study’s focus on media representation. Sources were coded according to the role attributed to them by journalists in each article, not according to all demographic characteristics they might hold. For instance, Vanessa Nakate (a female youth climate activist from Uganda) was coded as “youth” when quoted on youth-led movements and as “grassroots activist” when quoted as a protest organizer. This approach captures how media construct and deploy voiceswhich is precisely what the study investigates. We note that politicians, regardless of their national origin, would not be classified as marginalized voices given their institutional access and platform.

**3. Analytical Integration and Scope**

***Concern:*** *Bridge the gap between theory and data by linking findings to the three mechanisms of social capital.*

**Response:** Social capital theory serves as an interpretive framework in this study rather than a set of variables to be directly operationalized. Our content analysis documents the *outcomes* of differential social capitalspecifically, which voices gain media access and which are marginalizedrather than directly measuring the mechanisms themselves (institutional proximity, professional credibility, network embeddedness), which operate within journalistic production processes that precede the published articles we analyzed. We have added clarifying text at the start of the Discussion to make this interpretive relationship more explicit.

***Concern:*** *Move beyond counting to explain the “how” of exclusion.*

**Response:** The patterns we identified reflect these mechanisms at work: the dominance of political sources across all outlets reflects institutional proximity through established press offices and communication protocols; the prominence of credentialed scientists reflects how professional credibility functions as cultural capital translating into media access; and the consistency of source selection across ideologically different outlets reflects network embeddedness among elite actors. Directly observing these mechanisms would require newsroom ethnography or journalist interviews, which falls outside this study’s scope but represents a valuable direction for future research.

***Concern:*** *Chi-square tables must include actual counts (N) alongside percentages.*

**Response:** We thank the reviewer for this recommendation. All chi-square cross-tabulation tables have been standardized to include actual counts (N) alongside column percentages in the format N (%), consistent with reporting conventions in quantitative media research.

During the process of recomputing chi-square statistics directly from the raw dataset, several values were corrected. Notably, the chi-square test for source type distribution across publications yielded a non-significant result (χ²(18) = 21.641, p = 0.248), indicating that the four outlets did not differ significantly in the types of sources they cited. We have revised the relevant findings and discussion sections accordingly. Rather than undermining our argument, this finding strengthens it: all four publications, regardless of ideological orientation or geographic positioning, drew from essentially the same source hierarchy —politicians, scientists, and activists, suggesting that institutional sourcing norms reproduce elite access patterns across outlets. The corrected chi-square statistics for all variables are reported in the revised manuscript tables and in-text.

**4. References**

***Concern:*** *Update references more than 20 years old.*

**Response:** We have reviewed all older citations and updated where appropriate while retaining foundational works essential to the historical narrative and theoretical framework. Specifically:

• Domke (1997) has been replaced with Domke (2001), “Racial Cues and Political Ideology,” *Communication Research*, 28(6), 772–801.

• Entman (1993) has been removed as Entman (2007) is already cited.

• Moser (2010) has been replaced with Moser (2016), “Reflections on climate change communication,” *WIREs Climate Change*, 7(3).

• Schudson (2001) has been replaced with Schudson (2018), *Why Journalism Still Matters*, Cambridge: Polity Press.

Canonical works (e.g., Hall, 1995; Bourdieu, 1986; McCombs & Shaw, 1972) have been retained as they remain foundational to media studies and social capital scholarship. All references have been reformatted to Vancouver style per PLOS ONE requirements.

We trust these revisions address the points raised and have strengthened the manuscript considerably.

# REVIEWER #2

We thank the reviewer for the positive assessment of our manuscript and for the careful attention to detail.

***Concern:*** *Fix grammatical errors throughout.*

**Response:** We have conducted a thorough review of the manuscript and corrected all identified grammatical errors. The revised text has been reviewed for clarity, consistency, and adherence to academic writing conventions.

***Concern:*** *Tilt analysis more toward findings generally.*

**Response:** The analysis is structured around the findings themselves, with each research question addressed through direct engagement with the empirical results. We have reviewed the analytical sections to ensure the discussion remains grounded in the data while drawing appropriate theoretical connections.

# REVIEWER #3

Dear Dr. Wilfred O. Odoyo,

We appreciate your thoughtful engagement with our manuscript and the constructive suggestions for expanding its scope. Several of your recommendations point toward valuable directions for future research, and we have addressed each point below.

**Scope and Limitations**

***Concern:*** *Limited temporal scopeonly first week of COP26 analyzed.*

**Response:** We acknowledge this limitation explicitly in the manuscript. Analyzing only the summit’s first week may overlook narrative evolution during the second week when negotiations intensified and agreements were reached. A longitudinal comparison of pre-, during-, and post-event coverage would indeed enrich understanding of how media narratives develop and could constitute a valuable follow-up study.

***Concern:*** *Sample limited to Global North outletsmisses Southern perspectives.*

**Response:** This limitation is acknowledged in the manuscript. Our deliberate focus on UK and US outlets (Global North) aimed to examine how elite Western media construct climate voices, but this necessarily excludes Global South perspectives on COP26 coverage. The inclusion of The Herald provided a local Glasgow perspective to complement the national outlets. Comparative analysis incorporating Global South media would offer important insights and represents a priority for future research.

***Concern:*** *Social media excludedmisses alternative discourse spaces.*

**Response:** We acknowledge this gap in the manuscript. Our focus on mainstream print media was a deliberate methodological choice, as analyzing social media discourse (e.g., hashtag analysis on Instagram or X) would constitute a substantially different study with distinct sampling, coding, and analytical requirements. We note in the Discussion that social media platforms have introduced opportunities for marginalized voices to gain visibility and build alternative forms of social capital, though our findings suggest this has had limited impact on mainstream media representation.

***Concern:*** *No direct voices from marginalized communitiesrelies on media representation.*

**Response:** The reviewer correctly identifies that our study analyzes media representation rather than lived experience. This was a deliberate methodological choice: our research questions concern how mainstream media construct and deploy voices in climate discourse, not how marginalized communities experience or perceive that coverage. Interviews with affected communities would provide valuable complementary insights and could form the basis of future research examining the relationship between media representation and community perspectives.

**Analysis and Structure**

***Concern:*** *Gender analysis underdevelopedonly briefly mentioned in findings.*

**Response:** The paper did not set out to analyze any single characteristic of marginalized voices in depth (whether gender, race, age, geographic location, or role). Rather, our objective was to examine the overall representation and marginalization of these voices in aggregate. Developing a full gender analysis would require a dedicated study examining intersectional dimensions of climate voice representationa worthy endeavor that exceeds the scope of this paper but represents a promising direction for future research.

***Concern:*** *Discussion could engage more with “solutions framing”NYT’s renewable energy focus not fully explored.*

**Response:** We have a dedicated subsection on Solutions Framing that compares how each newspaper presents solutions, and the Discussion analyzes the implications. Our analysis notes that NYT’s overwhelming focus on renewable energy suggests a primarily technological framing that may crowd out equally important approachesconsumption reduction, structural economic change, climate justice and reparations, or adaptation strategies. Crucially, this technological framing included minimal marginalized voices as spokespeople, reinforcing our central argument about voice representation.

***Concern:*** *Embed figures/tables in the draft for cohesiveness.*

**Response:** All figures and tables are now embedded within the manuscript at their point of first reference, with additional detail added to enhance interpretability.

***Concern:*** *Conclusion could be more forward-lookingadd recommendations for journalists, educators, and policymakers.*

**Response:** We have expanded the concluding section to reinforce the interconnected nature of stakeholder responsibilities. The manuscript includes specific recommendations for media outlets (partnering with community organizations and Indigenous networks) and for individual journalists (dedicating beats to environmental justice, recruiting from marginalized communities, developing relationships beyond official channels). We have added text emphasizing that achieving more inclusive climate discourse requires structural transformation across institutions. We note that recommendations for educators, while valuable, fall outside the scope of a media content analysis study.

We are grateful for these suggestions, many of which point toward productive directions for future research on climate communication and voice representation.
