# Supplementary material for: Silenced and privileged voices in media discourses: Climate change and social capital
Source: PLoS One. 2026 Jul 1;21(7):e0350826. doi: 10.1371/journal.pone.0350826 (PMC13322496; doi:10.1371/journal.pone.0350826)
Supplement: Peer review history

# Peer review: Decision Letter 0

**DOI:** 10.1371/journal.pone.0350826.r001

**Authors:** Daniel Parkes

13 Jan 2026

PONE-D-25-60955

Silenced and Privileged Voices in Media Discourses: Climate Change and Social Capital

PLOS One

Dear Dr. Bashri,

Thank you for submitting your manuscript to PLOS ONE. After careful consideration, we feel that it has merit but does not fully meet PLOS ONE’s publication criteria as it currently stands. Therefore, we invite you to submit a revised version of the manuscript that addresses the points raised during the review process.

Please submit your revised manuscript by Feb 26 2026 11:59PM. If you will need more time than this to complete your revisions, please reply to this message or contact the journal office at plosone@plos.org. When you're ready to submit your revision, log on to https://www.editorialmanager.com/pone/ and select the 'Submissions Needing Revision' folder to locate your manuscript file.

Please include the following items when submitting your revised manuscript:

- A letter that responds to each point raised by the academic editor and reviewer(s). You should upload this letter as a separate file labeled 'Response to Reviewers'.
- A marked-up copy of your manuscript that highlights changes made to the original version. You should upload this as a separate file labeled 'Revised Manuscript with Track Changes'.
- An unmarked version of your revised paper without tracked changes. You should upload this as a separate file labeled 'Manuscript'.

If you would like to make changes to your financial disclosure, please include your updated statement in your cover letter. Guidelines for resubmitting your figure files are available below the reviewer comments at the end of this letter.

If applicable, we recommend that you deposit your laboratory protocols in protocols.io to enhance the reproducibility of your results. Protocols.io assigns your protocol its own identifier (DOI) so that it can be cited independently in the future. For instructions see: https://journals.plos.org/plosone/s/submission-guidelines#loc-laboratory-protocols. Additionally, PLOS ONE offers an option for publishing peer-reviewed Lab Protocol articles, which describe protocols hosted on protocols.io. Read more information on sharing protocols at https://plos.org/protocols?utm_medium=editorial-email&utm_source=authorletters&utm_campaign=protocols.

We look forward to receiving your revised manuscript.

Kind regards,

Daniel Parkes, PhD

Staff Editor

PLOS One

Journal Requirements:

When submitting your revision, we need you to address these additional requirements.

1.Please ensure that your manuscript meets PLOS ONE's style requirements, including those for file naming. The PLOS ONE style templates can be found at

https://journals.plos.org/plosone/s/file?id=wjVg/PLOSOne_formatting_sample_main_body.pdf and

https://journals.plos.org/plosone/s/file?id=ba62/PLOSOne_formatting_sample_title_authors_affiliations.pdf

2. Please note that PLOS One has specific guidelines on code sharing for submissions in which author-generated code underpins the findings in the manuscript. In these cases, we expect all author-generated code to be made available without restrictions upon publication of the work. Please review our guidelines at https://journals.plos.org/plosone/s/materials-and-software-sharing#loc-sharing-code and ensure that your code is shared in a way that follows best practice and facilitates reproducibility and reuse.

3. We note that your Data Availability Statement is currently as follows: All relevant data are within the manuscript and in Supporting Information files.

Please confirm at this time whether or not your submission contains all raw data required to replicate the results of your study. Authors must share the “minimal data set” for their submission. PLOS defines the minimal data set to consist of the data required to replicate all study findings reported in the article, as well as related metadata and methods (https://journals.plos.org/plosone/s/data-availability#loc-minimal-data-set-definition).

For example, authors should submit the following data:

- The values behind the means, standard deviations and other measures reported;

- The values used to build graphs;

- The points extracted from images for analysis.

Authors do not need to submit their entire data set if only a portion of the data was used in the reported study.

If your submission does not contain these data, please either upload them as Supporting Information files or deposit them to a stable, public repository and provide us with the relevant URLs, DOIs, or accession numbers. For a list of recommended repositories, please see https://journals.plos.org/plosone/s/recommended-repositories.

If there are ethical or legal restrictions on sharing a de-identified data set, please explain them in detail (e.g., data contain potentially sensitive information, data are owned by a third-party organization, etc.) and who has imposed them (e.g., an ethics committee). Please also provide contact information for a data access committee, ethics committee, or other institutional body to which data requests may be sent. If data are owned by a third party, please indicate how others may request data access.

4. Please upload a copy of Figures 1-3, to which you refer in your text on page 17 and 19. If the figure is no longer to be included as part of the submission please remove all reference to it within the text.

5. Please include a copy of Table 1-4 which you refer to in your text on page 15, 17 and 19.

6. Please include captions for your Supporting Information files at the end of your manuscript, and update any in-text citations to match accordingly. Please see our Supporting Information guidelines for more information: http://journals.plos.org/plosone/s/supporting-information.

7. If the reviewer comments include a recommendation to cite specific previously published works, please review and evaluate these publications to determine whether they are relevant and should be cited. There is no requirement to cite these works unless the editor has indicated otherwise.

[Note: HTML markup is below. Please do not edit.]

Reviewers' comments:

Reviewer's Responses to Questions

**Comments to the Author**

1. Is the manuscript technically sound, and do the data support the conclusions?

The manuscript must describe a technically sound piece of scientific research with data that supports the conclusions. Experiments must have been conducted rigorously, with appropriate controls, replication, and sample sizes. The conclusions must be drawn appropriately based on the data presented.

Reviewer #1: Partly

Reviewer #2: Partly

Reviewer #3: Yes

**********

2. Has the statistical analysis been performed appropriately and rigorously?

Reviewer #1: N/A

Reviewer #2: Yes

Reviewer #3: Yes

**********

3. Have the authors made all data underlying the findings in their manuscript fully available?

The PLOS Data policy requires authors to make all data underlying the findings described in their manuscript fully available without restriction, with rare exception (please refer to the Data Availability Statement in the manuscript PDF file). The data should be provided as part of the manuscript or its supporting information, or deposited to a public repository. For example, in addition to summary statistics, the data points behind means, medians and variance measures should be available. If there are restrictions on publicly sharing data—e.g. participant privacy or use of data from a third party—those must be specified.

Reviewer #1: Yes

Reviewer #2: Yes

Reviewer #3: Yes

**********

4. Is the manuscript presented in an intelligible fashion and written in standard English?

PLOS ONE does not copyedit accepted manuscripts, so the language in submitted articles must be clear, correct, and unambiguous. Any typographical or grammatical errors should be corrected at revision, so please note any specific errors here.

Reviewer #1: Yes

Reviewer #2: Yes

Reviewer #3: Yes

**********

5. Review Comments to the Author

Please use the space provided to explain your answers to the questions above. You may also include additional comments for the author, including concerns about dual publication, research ethics, or publication ethics. (Please upload your review as an attachment if it exceeds 20,000 characters)

Reviewer #1: Comments

While the study presents original research through a content analysis of media coverage during COP26, it must address several critical areas to meet the required standards for publication. Although the research utilizes a dual quantitative and qualitative approach, the methodology currently lacks the necessary rigor due to significant transparency gaps in the qualitative phase. The manuscript adheres to a standard academic structure; the reporting standards must be improved by providing more comprehensive detail in the "Methodology" and "Findings" sections to ensure the study is fully replicable by other researchers.

• The study exhibits a significant level of methodological vagueness, particularly regarding the qualitative phase of the research. While the quantitative inter-coder reliability is reported as strong at 0.87, the manuscript fails to detail the specific qualitative framework employed—such as Thematic Analysis or Critical Discourse Analysis—to "uncover depth" in the narratives. Without a clearly defined qualitative procedure or a description of the open-coding process, the interpretations risk appearing subjective rather than systematic, which undermines the scientific rigor required for replication.

• There is also a notable theoretical and empirical disconnect concerning the application of "Social Capital." Although the literature review provides an extensive discussion of Bourdieu’s Social Capital and its three mechanisms, the findings focus primarily on the frequency of citations rather than the functional application of these mechanisms. The analysis fails to demonstrate how institutional proximity or professional credibility specifically operated to exclude marginalized voices in the selected articles, leaving the theoretical framework underutilized in the actual results.

Required Revisions

1. Methodological Enhancements and Transparency. To meet publication standards, the authors must explicitly state the qualitative method used and provide a step-by-step description of how themes were derived beyond the pre-set categories. Additionally, the "Other" category must be broken down or summarized to identify what these frames actually represented. To ensure full transparency and compliance with PLOS ONE’s data policy, the authors are required to provide the raw coding data (such as Excel or SPSS sheets) as supplementary files.

2. Analytical Integration and Scope. The authors need to bridge the gap between theory and data by explicitly linking the discussion of findings to the three mechanisms of social capital mentioned in the introduction. The analysis should move beyond mere counting to explain the "how" of exclusion. Furthermore, all statistical reporting must be standardized; Chi-square tables must include actual counts (N) alongside percentages to allow for independent verification of statistical significance.

3. Clarification of Source Categorization. The manuscript requires a more precise definition of "marginalized" voices. The authors should clarify the hierarchy used when an individual fits multiple categories—for example, how a Global South representative who is also a politician was categorized—to ensure the data accurately reflects the exclusion of specific demographics versus professional roles.

4. Many old references, some more than 20 years old, were cited. I suggest the author(s) update some of these references with newer ones, especially those unrelated to the historical narrative of the topic.

Reviewer #2: The manuscript is good and publishable. The topic is topical considering the impact of climate change and war to mitigate it all over the world. But there is need to effect minor corrections regarding a few grammatical blunder noticed. The analysis should be tilted towards the finding generally.

Reviewer #3: 1. Limited temporal scope – Only first week of COP26 analyzed. Compare pre-, during, and post-event coverage to capture evolving naratives.

2. Sample limited to Global North outlets – Misses Southern perspectives. Global South newspaper need representation.

3. Social media excluded – Misses alternative discourse spaces. Social media is big in hashtags. Let's hear what they said in relation to COP26. (Instagram, X)

4. Gender analysis underdeveloped – Only briefly mentioned in findings.

5. Discussion could engage more with “solutions framing” – Findings show NYT focused on renewable energy, but implications are not fully explored.

6. No direct voices from marginalized communities – Relies on media representation, not lived experience. Consider adding interviews

7. Embed figures/tables referenced in the draft – Limits assessment of data presentation. (they are attached but would be more informative if embedded in the paper for ease of referencing and cohesiveness)

8. Conclusion could be more forward-looking – Ends somewhat abruptly. - add a 'recommendations' subsection for journalists, educators, and policymakers to diversify climate storytelling.

**********

6. PLOS authors have the option to publish the peer review history of their article (what does this mean?). If published, this will include your full peer review and any attached files.

If you choose “no”, your identity will remain anonymous but your review may still be made public.

**Do you want your identity to be public for this peer review?**    For information about this choice, including consent withdrawal, please see our Privacy Policy.

Reviewer #1: **Yes:**    Dr. Najm A. Alhatimi Aleessawi

Reviewer #2: No

Reviewer #3: **Yes:**    Wilfred O. Odoyo, PhD.

**********

[NOTE: If reviewer comments were submitted as an attachment file, they will be attached to this email and accessible via the submission site. Please log into your account, locate the manuscript record, and check for the action link "View Attachments". If this link does not appear, there are no attachment files.]

To ensure your figures meet our technical requirements, please review our figure guidelines: https://journals.plos.org/plosone/s/figures

You may also use PLOS’s free figure tool, NAAS, to help you prepare publication quality figures: https://journals.plos.org/plosone/s/figures#loc-tools-for-figure-preparation.

NAAS will assess whether your figures meet our technical requirements by comparing each figure against our figure specifications.

# Author comment: Author response to Decision Letter 1

**DOI:** 10.1371/journal.pone.0350826.r002

27 Feb 2026

RESPONSE TO REVIEWERS

REVIEWER #1

Dear Dr. Najm A. Alhatimi Aleessawi,

We are grateful for the time, care, and scholarly rigor you brought to your review of our manuscript. Your comments reflect a deep engagement with both the methodological and theoretical dimensions of the study, and we found your observations to be incisive and constructive. We have addressed each of your points below, with specific references to where revisions appear in the text.

1. Methodological Enhancements and Transparency

Concern: The manuscript fails to detail the specific qualitative framework employed.

Response: Our study employed inductive thematic analysis following Braun and Clarke’s (2006, 2021) frameworka well-established approach for media content research. To enhance clarity, we have added a dedicated Analytical Approach subsection to the methodology that details the six-phase process of theme development: familiarization, initial coding, searching for themes, reviewing themes, defining themes, and final synthesis.

Concern: The “Other” category must be broken down or summarized.

Response: The “Other” category represents frames that appeared only once or twice across the dataset and did not demonstrate sufficient patterning to constitute standalone themes. Retaining these under “Other” preserves analytic transparency rather than forcing isolated instances into ill-fitting categories. To address the reviewer’s concern, we have added concrete examples: content categorized as Other included local public, police, and emergency services (sources); travel logistics, wheelchair access, and speeches (themes); destruction of habitats, loss of culture, and impacts on animals (consequences); greenwashing, lack of transparency, and disagreement about facts (conflict); NGOs and community organisations (voices); lack of investment, awareness, and resource allocation (causes); and technology, parity and justice, and industry behaviour (solutions).

Concern: Provide raw coding data as supplementary files.

Response: The raw coding data has been deposited in a public repository. To preserve blind review, the DOI and access details have been provided to the editor and will be included in the published manuscript upon acceptance. The dataset has also been shared with the editor. Additionally, we have now embedded Tables 1–4 containing the full chi-square cross-tabulations in the manuscript.

2. Clarification of Source Categorization

Concern: The manuscript requires a more precise definition of marginalized voices.

Response: We have added a definition in the methodology section. For this study, marginalized voices refers to groups that face systematic barriers to media access and representation in climate discourse, typically lacking the institutional proximity, professional credibility, and network embeddedness that facilitate routine media access. These voices are marginalized in that they are pushed to the periphery of climate discoursetheir knowledge, experiences, and proposed solutions are less likely to be cited, less likely to frame media narratives, and consequently less likely to influence public understanding and policymaking, despite their lived expertise with climate impacts.

Concern: Clarify the coding hierarchy when an individual fits multiple categories.

Response: Our coding followed a role-based protocol consistent with the study’s focus on media representation. Sources were coded according to the role attributed to them by journalists in each article, not according to all demographic characteristics they might hold. For instance, Vanessa Nakate (a female youth climate activist from Uganda) was coded as “youth” when quoted on youth-led movements and as “grassroots activist” when quoted as a protest organizer. This approach captures how media construct and deploy voiceswhich is precisely what the study investigates. We note that politicians, regardless of their national origin, would not be classified as marginalized voices given their institutional access and platform.

3. Analytical Integration and Scope

Concern: Bridge the gap between theory and data by linking findings to the three mechanisms of social capital.

Response: Social capital theory serves as an interpretive framework in this study rather than a set of variables to be directly operationalized. Our content analysis documents the outcomes of differential social capitalspecifically, which voices gain media access and which are marginalizedrather than directly measuring the mechanisms themselves (institutional proximity, professional credibility, network embeddedness), which operate within journalistic production processes that precede the published articles we analyzed. We have added clarifying text at the start of the Discussion to make this interpretive relationship more explicit.

Concern: Move beyond counting to explain the “how” of exclusion.

Response: The patterns we identified reflect these mechanisms at work: the dominance of political sources across all outlets reflects institutional proximity through established press offices and communication protocols; the prominence of credentialed scientists reflects how professional credibility functions as cultural capital translating into media access; and the consistency of source selection across ideologically different outlets reflects network embeddedness among elite actors. Directly observing these mechanisms would require newsroom ethnography or journalist interviews, which falls outside this study’s scope but represents a valuable direction for future research.

Concern: Chi-square tables must include actual counts (N) alongside percentages.

Response: We thank the reviewer for this recommendation. All chi-square cross-tabulation tables have been standardized to include actual counts (N) alongside column percentages in the format N (%), consistent with reporting conventions in quantitative media research.

During the process of recomputing chi-square statistics directly from the raw dataset, several values were corrected. Notably, the chi-square test for source type distribution across publications yielded a non-significant result (χ²(18) = 21.641, p = 0.248), indicating that the four outlets did not differ significantly in the types of sources they cited. We have revised the relevant findings and discussion sections accordingly. Rather than undermining our argument, this finding strengthens it: all four publications, regardless of ideological orientation or geographic positioning, drew from essentially the same source hierarchy —politicians, scientists, and activists, suggesting that institutional sourcing norms reproduce elite access patterns across outlets. The corrected chi-square statistics for all variables are reported in the revised manuscript tables and in-text.

4. References

Concern: Update references more than 20 years old.

Response: We have reviewed all older citations and updated where appropriate while retaining foundational works essential to the historical narrative and theoretical framework. Specifically:

• Domke (1997) has been replaced with Domke (2001), “Racial Cues and Political Ideology,” Communication Research, 28(6), 772–801.

• Entman (1993) has been removed as Entman (2007) is already cited.

• Moser (2010) has been replaced with Moser (2016), “Reflections on climate change communication,” WIREs Climate Change, 7(3).

• Schudson (2001) has been replaced with Schudson (2018), Why Journalism Still Matters, Cambridge: Polity Press.

Canonical works (e.g., Hall, 1995; Bourdieu, 1986; McCombs & Shaw, 1972) have been retained as they remain foundational to media studies and social capital scholarship. All references have been reformatted to Vancouver style per PLOS ONE requirements.

We trust these revisions address the points raised and have strengthened the manuscript considerably.

REVIEWER #2

We thank the reviewer for the positive assessment of our manuscript and for the careful attention to detail.

Concern: Fix grammatical errors throughout.

Response: We have conducted a thorough review of the manuscript and corrected all identified grammatical errors. The revised text has been reviewed for clarity, consistency, and adherence to academic writing conventions.

Concern: Tilt analysis more toward findings generally.

Response: The analysis is structured around the findings themselves, with each research question addressed through direct engagement with the empirical results. We have reviewed the analytical sections to ensure the discussion remains grounded in the data while drawing appropriate theoretical connections.

REVIEWER #3

Dear Dr. Wilfred O. Odoyo,

We appreciate your thoughtful engagement with our manuscript and the constructive suggestions for expanding its scope. Several of your recommendations point toward valuable directions for future research, and we have addressed each point below.

Scope and Limitations

Concern: Limited temporal scope only first week of COP26 analyzed.

Response: We acknowledge this limitation explicitly in the manuscript. Analyzing only the summit’s first week may overlook narrative evolution during the second week when negotiations intensified and agreements were reached. A longitudinal comparison of pre-, during-, and post-event coverage would indeed enrich understanding of how media narratives develop and could constitute a valuable follow-up study.

Concern: Sample limited to Global North outlets misses Southern perspectives.

Response: This limitation is acknowledged in the manuscript. Our deliberate focus on UK and US outlets (Global North) aimed to examine how elite Western media construct climate voices, but this necessarily excludes Global South perspectives on COP26 coverage. The inclusion of The Herald provided a local Glasgow perspective to complement the national outlets. Comparative analysis incorporating Global South media would offer important insights and represents a priority for future research.

Concern: Social media excluded misses alternative discourse spaces.

Response: We acknowledge this gap in the manuscript. Our focus on mainstream print media was a deliberate methodological choice, as analyzing social media discourse (e.g., hashtag analysis on Instagram or X) would constitute a substantially different study with distinct sampling, coding, and analytical requirements. We note in the Discussion that social media platforms have introduced opportunities for marginalized voices to gain visibility and build alternative forms of social capital, though our findings suggest this has had limited impact on mainstream media representation.

Concern: No direct voices from marginalized communities relies on media representation.

Response: The reviewer correctly identifies that our study analyzes media representation rather than lived experience. This was a deliberate methodological choice: our research questions concern how mainstream media construct and deploy voices in climate discourse, not how marginalized communities experience or perceive that coverage. Interviews with affected communities would provide valuable complementary insights and could form the basis of future research examining the relationship between media representation and community perspectives.

Analysis and Structure

Concern: Gender analysis underdevelopedonly briefly mentioned in findings.

Response: The paper did not set out to analyze any single characteristic of marginalized voices in depth (whether gender, race, age, geographic location, or role). Rather, our objective was to examine the overall representation and marginalization of these voices in aggregate. Developing a full gender analysis would require a dedicated study examining intersectional dimensions of climate voice representationa worthy endeavor that exceeds the scope of this paper but represents a promising direction for future research.

Concern: Discussion could engage more with “solutions framing”NYT’s renewable energy focus not fully explored.

Response: We have a dedicated subsection on Solutions Framing that compares how each newspaper presents solutions, and the Discussion analyzes the implications. Our analysis notes that NYT’s overwhelming focus on renewable energy suggests a primarily technological framing that may crowd out equally important approachesconsumption reduction, structural economic change, climate justice and reparations, or adaptation strategies. Crucially, this technological framing included minimal marginalized voices as spokespeople, reinforcing our central argument about voice representation.

Concern: Embed figures/tables in the draft for cohesiveness.

Response: All figures and tables are now embedded within the manuscript at their point of first reference, with additional detail added to enhance interpretability.

Concern: Conclusion could be more forward-looking add recommendations for journalists, educators, and policymakers.

Response: We have expanded the concluding section to reinforce the interconnected nature of stakeholder responsibilities. The manuscript includes specific recommendations for media outlets (partnering with community organizations and Indigenous networks) and for individual journalists (dedicating beats to environmental justice, recruiting from marginalized communities, developing relationships beyond official channels). We have added text emphasizing that achieving more inclusive climate discourse requires structural transformation across institutions. We note that recommendations for educators, while valuable, fall outside the scope of a media content analysis study.

We are grateful for these suggestions, many of which point toward productive directions for future research on climate communication and voice representation.

Attachment Submitted filename: Response to Reviewers.docx

# Peer review: Decision Letter 1

**DOI:** 10.1371/journal.pone.0350826.r003

**Authors:** Carla Pegoraro

20 May 2026

Silenced and Privileged Voices in Media Discourses: Climate Change and Social Capital

PONE-D-25-60955R1

Dear Dr. Bashri,

We’re pleased to inform you that your manuscript has been judged scientifically suitable for publication and will be formally accepted for publication once it meets all outstanding technical requirements.

Within one week, you’ll receive an e-mail detailing the required amendments. When these have been addressed, you’ll receive a formal acceptance letter and your manuscript will be scheduled for publication.

An invoice will be generated when your article is formally accepted. Please note, if your institution has a publishing partnership with PLOS and your article meets the relevant criteria, all or part of your publication costs will be covered. Please make sure your user information is up-to-date by logging into Editorial Manager at Editorial Manager® and clicking the ‘Update My Information' link at the top of the page. For questions related to billing, please contact billing support.

If your institution or institutions have a press office, please notify them about your upcoming paper to help maximize its impact. If they’ll be preparing press materials, please inform our press team as soon as possible -- no later than 48 hours after receiving the formal acceptance. Your manuscript will remain under strict press embargo until 2 pm Eastern Time on the date of publication. For more information, please contact onepress@plos.org.

Kind regards,

Carla Pegoraro

Staff Editor

PLOS One

Additional Editor Comments (optional):

Reviewers' comments:

Reviewer's Responses to Questions

**Comments to the Author**

1. If the authors have adequately addressed your comments raised in a previous round of review and you feel that this manuscript is now acceptable for publication, you may indicate that here to bypass the “Comments to the Author” section, enter your conflict of interest statement in the “Confidential to Editor” section, and submit your "Accept" recommendation.

Reviewer #1: All comments have been addressed

**********

2. Is the manuscript technically sound, and do the data support the conclusions?<br/><br/>The manuscript must describe a technically sound piece of scientific research with data that supports the conclusions. Experiments must have been conducted rigorously, with appropriate controls, replication, and sample sizes. The conclusions must be drawn appropriately based on the data presented.

Reviewer #1: Yes

**********

3. Has the statistical analysis been performed appropriately and rigorously?

Reviewer #1: Yes

**********

4. Have the authors made all data underlying the findings in their manuscript fully available?<br/><br/>The PLOS Data policy requires authors to make all data underlying the findings described in their manuscript fully available without restriction, with rare exception (please refer to the Data Availability Statement in the manuscript PDF file). The data should be provided as part of the manuscript or its supporting information, or deposited to a public repository. For example, in addition to summary statistics, the data points behind means, medians and variance measures should be available. If there are restrictions on publicly sharing data—e.g. participant privacy or use of data from a third party—those must be specified.

Reviewer #1: Yes

**********

5. Is the manuscript presented in an intelligible fashion and written in standard English?<br/><br/>PLOS ONE does not copyedit accepted manuscripts, so the language in submitted articles must be clear, correct, and unambiguous. Any typographical or grammatical errors should be corrected at revision, so please note any specific errors here.

Reviewer #1: Yes

**********

6. Review Comments to the Author<br/><br/>Please use the space provided to explain your answers to the questions above. You may also include additional comments for the author, including concerns about dual publication, research ethics, or publication ethics. (Please upload your review as an attachment if it exceeds 20,000 characters)

Reviewer #1: if it is suitable to the template, add a conclusion at the end of the paper (before the references)..

**********

7. PLOS authors have the option to publish the peer review history of their article (what does this mean?). If published, this will include your full peer review and any attached files.

If you choose “no”, your identity will remain anonymous but your review may still be made public.

**Do you want your identity to be public for this peer review?**    For information about this choice, including consent withdrawal, please see our Privacy Policy.

Reviewer #1: **Yes:**    Dr. Najm A. Kh. Alhatimi Aleessawi

**********

# Editor report: Acceptance letter

**DOI:** 10.1371/journal.pone.0350826.r004

**Authors:** Carla Pegoraro

PONE-D-25-60955R1

PLOS One

Dear Dr. Bashri,

I'm pleased to inform you that your manuscript has been deemed suitable for publication in PLOS One. Congratulations! Your manuscript is now being handed over to our production team.

At this stage, our production department will prepare your paper for publication. This includes ensuring the following:

* All references, tables, and figures are properly cited

* All relevant supporting information is included in the manuscript submission,

* There are no issues that prevent the paper from being properly typeset

You will receive further instructions from the production team, including instructions on how to review your proof when it is ready. Please keep in mind that we are working through a large volume of accepted articles, so please give us a few days to review your paper and let you know the next and final steps.

Lastly, if your institution or institutions have a press office, please let them know about your upcoming paper now to help maximize its impact. If they'll be preparing press materials, please inform our press team within the next 48 hours. Your manuscript will remain under strict press embargo until 2 pm Eastern Time on the date of publication. For more information, please contact onepress@plos.org.

You will receive an invoice from PLOS for your publication fee after your manuscript has reached the completed accept phase. If you receive an email requesting payment before acceptance or for any other service, this may be a phishing scheme. Learn how to identify phishing emails and protect your accounts at https://explore.plos.org/phishing.

If we can help with anything else, please email us at customercare@plos.org.

Thank you for submitting your work to PLOS ONE and supporting open access.

Kind regards,

PLOS ONE Editorial Office Staff

on behalf of

Dr Carla Pegoraro

Staff Editor

PLOS One
